# Health Tract, No. 12

**Published:** 1865

**Authors:** 


### HEALTH TRACT, NO. 12.

### HOW TO WALK.

If you wish to be aided in securing this habitual carriage of body, accustom yourself while walking to carry the hands behind you, one grasping the opposite wrist. Englishmen are admired the world over for their full chests, and broad shoulders, and sturdy frames, and manly bearing. This position of body is a favorite with them, in the simple promenade, in the garden or gallery, in attending ladies along a crowded street, in standing on the street, or in places of public worship.

Our young men seem to be in elysium when they can walk arm-in-arm with their divinities. Now, young gentlemen, you will be hooked on quite soon enough, without anticipating your captivity. While you are free, *walk right* in ALL WAYS; and when you are able, get a *manly* carriage; take our word for it, that it is the best way in the world to *secure* the affectionate respect of the woman you marry. Did you ever know any girl worth having, who could or would wed a man who mopes about with his eyes on the ground, making of his whole body the segment of a circle bent the wrong way? Assuredly, a woman of strong points, of striking characteristics, admires, beyond a handsome face, the whole carriage of a man. Erectness, being the representative of courage and daring, is that which makes a *man of presence* in the hour of impending danger or peril.

### Walking or Sleeping with the Mouth open.

There is one rule which should be strictly observed by all in taking exercise by walking — as the very best form in which it can be taken by both the young and the able-bodied of all ages — and that is, *never to allow the action of respiration or breathing to be carried on through the mouth.* The nasal passages are clearly the medium through which respiration was, by our Creator, designed to be carried on. "God breathed into man's nostrils the breath of life," previous to his becoming a living creature.

The difference in the exhaustion of strength by a long walk with the mouth firmly and resolutely closed, and respiration carried on through the nostrils instead of through the mouth, cannot be conceived as possible by those who have never tried the experiment. Indeed, this mischievous and really unnatural habit of carrying on the work of inspiration and expiration through the mouth, instead of through the nasal passages, is the true origin of almost all diseases of the throat and lungs, *bronchitis*, *congestion*, *asthma*, and even *consumption* itself.

That excessive perspiration to which some individuals are so liable in their sleep, and which is so weakening to the body, is solely the effect of such persons sleeping with their mouths unclosed. And the same exhaustive results arise to the animal system from walking with the mouth open, instead of — when not engaged in conversation — preserving the lips in a state of firm but quiet compression. Children should never be allowed to sleep, stand, or walk, with their mouths open; for, besides the vacant appearance it gives to the countenance, it sometimes causes *coughs*, *colds*, and *sore throats*.

